# Workplace burnout and health issues among Colombian correctional officers

**DOI:** 10.1371/journal.pone.0211447

**Published:** 2019-02-12

**Authors:** Sergio A. Useche, Luis V. Montoro, José I. Ruiz, César Vanegas, Jaime Sanmartin, Elisa Alfaro

**Affiliations:** 1 INTRAS—Faculty of Psychology, University of Valencia, Valencia, Spain; 2 Laboratory of Psychology and Law, National University of Colombia, Bogotá, Colombia; 3 National Penitentiary School, National Penitentiary and Prison Institute, Funza, Colombia; 4 Faculty of Medicine, University of Valencia, Valencia, Spain; Universite Cote d'Azur, FRANCE

## Abstract

**Introduction:**

Correctional employees typically work under adverse conditions that may enhance the occurrence of different negative psychological states. Burnout constitutes a high-risk phenomenon that may affect people’s physical/mental health and welfare, especially in vulnerable occupational groups.

**Objectives:**

The aim of this study was to characterize the burnout profile of correctional officers, and to associate their burnout profile with health issues and lifestyle factors.

**Methods:**

The full sample was composed of 219 Colombian correctional officers with a mean age of 30.18 years. A questionnaire composed of three sections was employed: demographic data, burnout, and health information.

**Results:**

A high proportion of participants reported burnout indicators, also significantly correlated to their health indicators and lifestyle factors. Cluster analyses were used in order to characterize the burnout/age (model A) and burnout/age/psychological disturbance (model B) profiles of correctional officers. Furthermore, significant differences were found when comparing frequencies of alcohol consumption and physical exercise (lifestyle indicators) and perceived social support of officers depending on their profile.

**Conclusions:**

the discussion focused on the negative impact of burnout on health, and on the importance of strengthening occupational programs aimed at reducing the impact of hazardous working conditions that contribute to the development of burnout, and to the arise different mid and long-term health complains among correctional workers.

## Introduction

In the scientific literature, the concept of *Burnout* has been widely theorized as a phenomenon which mainly arises as a response to a chronic exposure to work-related stressors [1], and which is composed of three main dimensions: first, the *emotional exhaustion*, defined as the emotional manifestation of chronic fatigue or stress; secondly, *depersonalization* or *cynicism*, that is the dimension of interpersonal context surrounding a burnout which implies negative interactions with (e.g.) supervisors, co-workers, users; and, third, a substantial reduction in the perception of *professional accomplishment* at the workplace [2].

Correctional facilities present some typical adverse operational conditions for the workers’ health, safety and welfare, since the job is usually characterized by, for instance, the presence of demanding and hazardous working conditions [3], high rates of physical and mental fatigue, risk of infectious diseases, irregular work shifts [4] and low rates of financial and professional rewards as a result of the efforts made [5], all of it systematically contributing to the decrease of the employees’ quality of life and professional accomplishment.

Finally, several studies have agreed on the fact that, in the case of prison staff (and mostly correctional officers) and other highly vulnerable groups, positive and significant correlations between emotional exhaustion and depersonalization (also known as cynicism) have been homogeneously found in various cases, at the same time as professional efficacy generally correlates negatively with the other two subscales of Maslach’s Burnout Inventory, or MBI [1,6].

### Work environment characteristics and burnout in occupational groups

Specific physical and environmental job features, in which some occupational activities take place, clearly predict the predisposition of subjects to suffer from or present symptoms related to burnout, especially in service-related jobs [7–9]; the same happens for some individual factors that may predict or trigger the psychosocial risk at work. In this sense, accumulated evidence has demonstrated the existence of higher rates of objective stressors and lack of social support, as well as elevated rates of unfavorable outcomes (e.g. absenteeism, occupational incidents), in certain key groups of workers, among which we find health service providers [8,10–12] caregivers [13], professional drivers [14,15] and correctional officers [16,17].

Burnout has also been widely associated with different negative organizational outcomes, such as absenteeism and high turnover/intention to quit [18,19]. Previous researches on the field of the intervention have found that the continuous evaluation and improvement of the task and the socio-emotional support from family and co-workers are relevant issues for the management and prevention of burnout [20–22]. Moreover, the burden of a workplace burnout consequences, in terms of absenteeism and lack of productivity for organizations, is considered substantially high, and it is globally estimated around USD300 billion per year [23].

According to the evidence, workers in different industries that experience burnout symptoms normally require the occupational implementation of programs and/or complex organizational strategies [6], in order to improve their way of coping with several comorbid factors, such as job stress [24,25], chronic fatigue and sleep problems [26], addictive behaviors [27,28], and different mid/long-term health complains [29,30].

### Burnout in prison staff

Some studies conducted in different countries have documented the high prevalence of burnout symptoms among correctional officers. For instance, Harizanova and Tamovska [31] found that, in the case of Bulgarian correctional officers, up to 74.5% may present cases of burnout. In general, high rates of workload positively influence the causation of emotional exhaustion and depersonalization; in contrast, a high level of job decisions latitude negatively influences the emotional exhaustion, while it is positively associated with worker’s individual accomplishment [32].

Furthermore, keeping in mind the different occupational characteristics that may contribute to the development of a burnout [33], in the specific case of correctional contexts the burnout symptomatology does not only affect guards, but potentially the entire prison staff [6], including professionals in the fields of mental health and penitentiary treatment [34]. Moreover, workplace burnout can affect the attitude towards the inmates and therefore substantially decrease the workers’ performance in tasks related to the adequate provision of treatment services and security tasks in penitentiary facilities [35]. Prison staff also deal with a documented and potentially hazardous characteristic implied in the task: public service stress. According to the evidence, burnout in workers performing occupational activities related to the public service and to the care of others may develop stress and stress-related absenteeism, and also burnout symptomatology, in relatively brief periods of active service [36]. Specifically, prison staff tend to manifest burnout symptoms during the first 5 years of service at their workplace. Furthermore, individuals with higher education and lower social support outside work tend to have higher levels of burnout [31]. In addition, Oliveira et al. [33] found that correctional employees with 10 years or more of tenure tend to present higher values of emotional exhaustion and lower rates of professional efficacy.

### Burnout and health complains of correctional officers

Workplace burnout has been largely associated with negative health outcomes of workers, such as: anxious and depressive disorders [36–38], sleep problems [39–41], chronic headaches [42], gastrointestinal illness [43], hypertension [44], muscle and pain-related issues [42], acute and chronic fatigue [45,46] and, especially in the case of the most vulnerable occupational groups and shift-workers [4,11], a progressively lower job performance and goal accomplishment [47].

Correctional officers have been proved to be more exposed to adverse psychosocial factors at work and to present poor results in what concerns their general mental health, when compared to diverse samples of workers employed in other fields [29,48]; they report more health problems, such as psychological distress [49], low self-esteem [48], post-traumatic stress disorder (PTSD) [50], and different issues related to physical health, such as ischemic heart disease [51], obesity [52], and diverse injuries derived from the execution of their working tasks [50]. Finally, some studies have documented worrying rates of prevalence of different addictive behaviors within this workforce, which seem to be related to the presence of work stressors, violence and burnout at the workplace. Some of the addictive behaviors are alcohol consumption, smoking and abuse of psychotropic drugs [28,53].

### ‘Usually together, but separately studied’: Gaps in the literature on burnout and health of prison staff

Although recent studies have addressed the high prevalence of both burnout and health complains among correctional officers, the study of these two spheres has been carried out usually separately. On the one hand, a great amount of the empirical researches exploring burnout on prison staff have focused on its work-related antecedents, dynamics and consequences, such as sick leave and absenteeism [54–56], and its relationship to work stress [57]. In the Colombian context, only two applied studies in the field have been published to the date in indexed journals [58,59]. On the other hand, researches addressing occupational health on prison staff members have contributed to describe different adverse physical and mental health outcomes, but scarcely relating them to burnout [56]. As for Colombian correctional officers, only two previous researches on this regard were found through a literature review, both describing a high risk of suffering infectious diseases, such as tuberculosis, and determining that the risk of this kind of illnesses could be up to 20 times greater for prison staff when compared to the general population [60,61]. No further research on other professional health issues of correctional officers was found during the literature review.

Moreover, only few studies such as those performed by Neveu [62] with French prison guards, and by Reeves [63] with American ones, tried to relate burnout with health outcomes, finding interesting relationships between both spheres, and suggesting the need of performing new researches in this regard. In this sense, both the current evidence and the need of deeper exploring these relationships in prison staff. contributed to develop the study objectives of this research. In practical terms, the main basis and essential contribution of this article is to generate a major understanding on the relationship between burnout and health in this vulnerable workforce.

### Objectives and hypotheses

The objectives of this study were, first, to characterize the burnout profile of correctional officers, and to establish relationships between their burnout profiles, health and lifestyle indicators.

Regarding study hypotheses, and bearing in mind the previous evidences addressed in the introduction of this article and, in accordance to the study objectives, it was hypothesized that:

1. Correctional officers will present differential burnout profiles linked to demographic factors and mental health indicators.
2. Participants with high-burnout profiles will show less favorable health and lifestyle outcomes, principally those related to occupational diseases, alcohol consumption and a less healthy lifestyle.

## Methods

### Sample

The sample was composed of *n* = 219 correctional officers (9% women and 91% men) between 18–53 years of age, with a mean age of $\bar{x}$ = 30.18 (*SD* = 6.07) years. All participants were active correctional officers working in four different Colombian penitentiary institutions under the administration of the National Penitentiary and Prison Institute (INPEC). Regarding their official ranks, 2.8% were trainees/cadets; 89.8% of them correction officer recruits; 2.3% of them were distinguished/senior correction officers, and 5.1% were inspector/chief prison officers. It is important to remark that, according to the current data protection laws and the will of the syndicates, they were not obligated to provide this information (i.e. officer rank), so the presented percentages correspond to the valid data (*n =* 176; 80.4% of the sample). As for they work tenure (highly correlated to age, with *σ =* .94**), 25.5% of them had 5 or less years of service; 23.6% 6 to 10; 33.5% 11 to 15; 14.2% 16 to 20, and 3.3% had more than 20 years of activity as correctional workers. Finally, and regarding their educational level, 10.04% (*n =* 22) had attended university, 37.9% (n = 83) had technical studies, and the remaining 52.06% (n = 114) of them only had secondary studies as their highest educational level, being secondary studies the minimum standard required for this type of job.

### Procedure, design and ethics

Participants completed the paper questionnaire at the facilities of the penitentiary institutions that had previously agreed to participate in the study. The study design and protocols were analyzed and approved by the Research Committee in Ethics of the University of Valencia (IRB number H1517828861658). Furthermore, the anonymity of the participants was guaranteed during the whole survey, and the fact that the data would only be used for research purposes was especially emphasized. An informed consent statement was used, signed by both parties before the participants answered the questionnaire.

Surveys were completed by 219 officers out of 250 initially receiving the invitation to participate, being the response rate approximately 88%. Additionally, considering the low rate of women (*n* = 24; 9% of sample), that also reflects their low participation in this occupational group, gender comparisons were not conducted; this was due to both the disproportionality of participants according to gender, and to the low statistical representativeness of this very small sub-sample.

### Description of the questionnaire

The questionnaire was administrated in Spanish, and consisted of three sections. In the first section, demographic variables (age, education, socio-economical strata) and descriptive working data (e.g. city of the penitentiary, hierarchical rank) were collected.

The second section included the Spanish version of the *Maslach’s Burnout Inventory* (MBI) [64–66]. This questionnaire consists of 22 items [0–6 Likert-scale] grouped in three sub-scales: emotional exhaustion (9 items, α = [0.88–0.90]), depersonalization/cynicism (5 items, α = [0.65–0.75]), and professional efficacy (8 items, α = [0.78–0.82]) [14,67,68].

Finally, the third part consisted of questions about issues related to health and social support, based on:

1. The 12-item version of General Health Questionnaire (GHQ) [69], for measuring Psychological Disturbance (PD) (α = 0.73 once negative items were reversed), and the 9-item version of the Perceived Social Support Scale (SPSS or EPAS) [70] for assessing the Subjective Social Support (SS) (α = 0.64) of correctional officers.
2. Self-report health indicators: height/weight (used to build BMI indicators), and the following self-reported markers of physical health: Do you smoke (Yes/No+Frequency)? Do you make physical exercise (Yes/No+Frequency)? Do you suffer from a) hypertension, b) high cholesterol or c) cardiovascular diseases, c) diabetes, d) ergonomic illnesses, and e) other important illness(es)?

### Data processing (statistical analysis)

For this study, descriptive analyses (measures of frequencies and central tendency) and correlational crossings were performed first, in order to describe the burnout profile and characterize the prevalence of health-related factors in correctional officers. In addition, Chi-square (χ2) analyses were performed in order to establish potential statistical associations between categorical study variables. After conducting normality tests and testing basic parameters, One-way ANOVA and Post-Hoc (Tukey) tests were used respectively to characterize and compare the mean scores of continuous study variables. Furthermore, Two-Step Cluster analyses were used for characterizing the job strain/burnout profile of prison guards. The sum of scores of emotional exhaustion, cynicism and professional efficacy (reversed score), and demographic characteristics were used as clustering variables. For the second model, the indicator of Psychological Disturbance (PD) was included. Once the data was obtained, the relevant statistical analyses were carried out using the Statistical Package for the Social Sciences (SPSS), version 23.0.

## Results

### Descriptive data

The scales’ descriptive statistics for the study are presented in Table 1. In comparison with previous studies, the mean/item score for emotional exhaustion (EE) (${\bar{x}}_{/\mathrm{item}}$ = 2.49), depersonalization or cynicism (CY) (${\bar{x}}_{/\mathrm{item}}$ = 1.77), and professional efficacy (PE) (${\bar{x}}_{/\mathrm{item}}$ = 4.45) of Colombian correctional officers are relatively lower for EE and CY, and higher in the case of PE, if compared to other Colombian occupational groups previously studied: a) professional drivers (${\bar{x}}_{/\mathrm{item}}$ of EE = 3.20, ${\bar{x}}_{/\mathrm{item}}$ of CY = 2.57 and ${\bar{x}}_{/\mathrm{item}}$ of PE = 4.33, in a 0–6 scale) [14], and b) university professors (estimated in ${\bar{x}}_{/\mathrm{item}}$ of EE = 2.9 and $\bar{x}/\mathrm{item}$ of CY = 2.1 ${\bar{x}}_{/\mathrm{item}}$ of PE = 4.41, in a 0–6 scale) [68].

**Table 1:** Descriptive results and pearson’s bivariate correlations between study variables.

No.	Variable	Mean	SD	2	3	4	5	6	7	8	9	10
*Demographic data*												
**1**	Age	3.18	6.07	.168*	.299**	.099	.001	.119	.044	.485**	-.208**	-.135
**2**	Socio-economical Strata (1–6)	2.19	.561	-	.273**	.047	-.003	-.062	-.056	.110	.003	-.086
**3**	Officer Rank				-	-.092	-.056	.172*	.039	.081	-.078	-.036
*MBI Factors*												
**4**	Emotional Exhaustion	22.37	12.20			-	.571**	-.267**	.431**	.018	.078	-.245**
**5**	Cynicism	8.82	6.18				-	-.256**	.230**	.152	.103	-.171*
**6**	Professional Efficacy	35.62	8.95					-	-.285**	-.084	-.151*	.031
*Health Indicators*												
**7**	Psychological Disturbance (GHQ)	9.92	4.47						-	.062	.224**	-.152*
**8**	Body Mass Index (BMI)	25.65	3.35							-	-.197*	-.102
**9**	Alcohol Consumption (Frequency)	1.26	.48								-	.119
**10**	Physical Exercise (Frequency)	2.27	7.88									-

* Correlation is significant at the 0.05 level (2-tailed).

** Correlation is significant at the 0.01 level (2-tailed).

### Correlation analysis

The correlation analysis (see Table 1) allowed us to establish the measures of positive and significant association between emotional exhaustion (EE) and cynicism (CY), such as with psychological disturbance (PD) as a standard indicator of the 12-item version of GHQ. In other words, higher scores in EE are related to higher rates of CY and PD. Furthermore, we found a negative significant association between EE and the frequency of performance of physical exercise, being EE scores higher for correctional officers with lower frequencies of exercise, and vice versa. The same correlations also apply for CY, being the significance and direction of these associations identical in the case of EE.

Referring to the professional efficacy (PE), a set of significant negative associations was found between PE scores and EE, CY, PD, and the frequency of alcohol consumption. Another important result to highlight is the fact that the rank of the officers is significantly associated [+] with their scores on professional efficacy. In other words, the higher is the rank, a greater perceived accomplishment at job is observed among them.

Regarding the demographic data and health-related issues of correctional officers, significant correlations were found between age, Body Mass Index (BMI) [+] and frequency of alcohol consumption [–]. Finally, PD were found to be significantly associated with alcohol consumption [+] and physical exercise [–].

### Prevalence of health issues

Based on the data provided by the correctional officers who took part in the study and using dichotomous indicators about their potential suffering from some specific diseases that tend to affect workers engaged in demanding works, it was determined that there is a prevalence of relevant physical illnesses and some health-related lifestyle habits, such as smoking, consuming alcohol and doing exercise. These results are shown in Table 2.

**Table 2:** Prevalence of self-reported health issues and lifestyle factors.

Health condition/habit	*n* (valid)	Yes		No	
		Frequency	Percentage (valid)	Frequency	Percentage (valid)
Hypertension	200	8	4.0%	192	96.0%
High Cholesterol	213	24	11.3%	189	88.7%
Cardiovascular Disease	203	8	3.9%	195	96.1%
Diabetes	204	4	2.0%	200	98.0%
Ergonomic Illnesses	203	52	2.6%	151	74.4%
Other Relevant Illness	203	19	9.4%	184	90.6%
Smoking	195	33	16.9%	162	83.1%
Alcohol Consumption	193	151	78.2%	42	21.8%
Physical Exercise	196	102	52.0%	94	48.0%
Overweight	158	96	60.8%	62	39.2%

It is worth focusing on a set of observed high percentages related to correctional officers´ health: 78.2% of them regularly consume alcohol; 60.8% of them is overweight; 16.9% have the habit of smoking and 11.3% have been diagnosed with high cholesterol. It is at least worrying that only approximately half of the sample (52%) has the habit of doing physical exercise, considering that their job is characterized by a large amount of physical demands. Finally, self-reported cardiovascular disease and diabetes mellitus rates are similar to the ones presented by the general population in this mean range of age.

### Categorical analysis

Statistical correspondences or associations were found in the case of the following couples of categorical variables: first, between the fact of being in the group with the highest scores of emotional exhaustion (EE) and presenting/reporting any ergonomic illness (*X*^*2*^_(1,203)_ = 9.182;p = 0.002); and secondly, significant associations were found between the fact of belonging to the group with the highest scores of cynicism (CY) and reporting to suffer from a) hypertension (*X*^*2*^_(1,200)_ = 5.347;p = 0.021), and b) ergonomic illness (*X*^*2*^_(1,203)_ = 5.301;p = 0.02). Finally, once built up an additional dichotomous variable for statistically comparing the prevalence of health problems according to the median distribution of the sample for “Burnout” (full score), significant associations were found with hypertension (*X*^*2*^_(1,199)_ = 4.814;p = 0.030) and ergonomic illnesses (*X*^*2*^_(1,202)_ = 6.408;p = 0.009), being in both cases the highest frequencies for officers over the sample median of Burnout.

### Differences in the psychological disturbance (PD) indicator

Through the One-way Analysis of Variance (ANOVA) we found a set of significant differences between correctional officers who presented different values in terms of psychological disturbance (PD). Specifically, the sample was divided in two groups, taking the median/percentile 50 as the cut-off point in order to determine whether there were differential rates in terms of burnout indicators. In brief, it was found that correctional officers presenting higher scores of PD have significantly higher mean values of EE (*F*_(1,217)_ = 24.426;p<0.001) and CY (*F*_(1,217)_ = 18.792;p<0.001) than prison guards who reported lower scores in PD. Also, it was found that correctional officers in the group of lower scores in PD presented higher mean values of PE (*F*_(1,217)_ = 11.449;p<0.001), as shown in Table 3.

**Table 3:** Mean comparisons (ANOVA) of MBI factors between groups of correctional officers*.

Variable/Group	Lower scores			Higher scores			ANOVA		
	Mean	SD	Std Error	Mean	SD	Std Error	F	df	Sig
Emotional Exhaustion (EE)	8.51	3.95	0.37	11.45	4.51	0.44	26.42	1, 217	*p<*0.001
Cynicism (CY)	8.71	4.07	0.38	11.23	4.53	0.44	18.79	1, 217	*p<*0.001
Professional Efficacy (PE)	10.89	4.08	0.39	8.90	4.64	0.45	11.44	1, 217	*p =* 0.001

*Factor: Group (Higher/Lower) according to P50 of Psychological Disturbance (PD).

### Two-step cluster analysis and mean comparisons

Considering that -according to the MBI approach- Burnout as a global phenomenon constitutes a combination of high emotional exhaustion (EE) and cynicism (CY) together with lower rates of professional efficacy, we created an unique variable based on the sum of these three sub-scales, being the total score for Burnout = EE score + CY score + reversed PE score, with the objective of combining burnout profiles and scores reported in other relevant variables, such as age and mental health indicators (i.e. psychological disturbance).

#### Cluster Model-A

**Fig 1:**
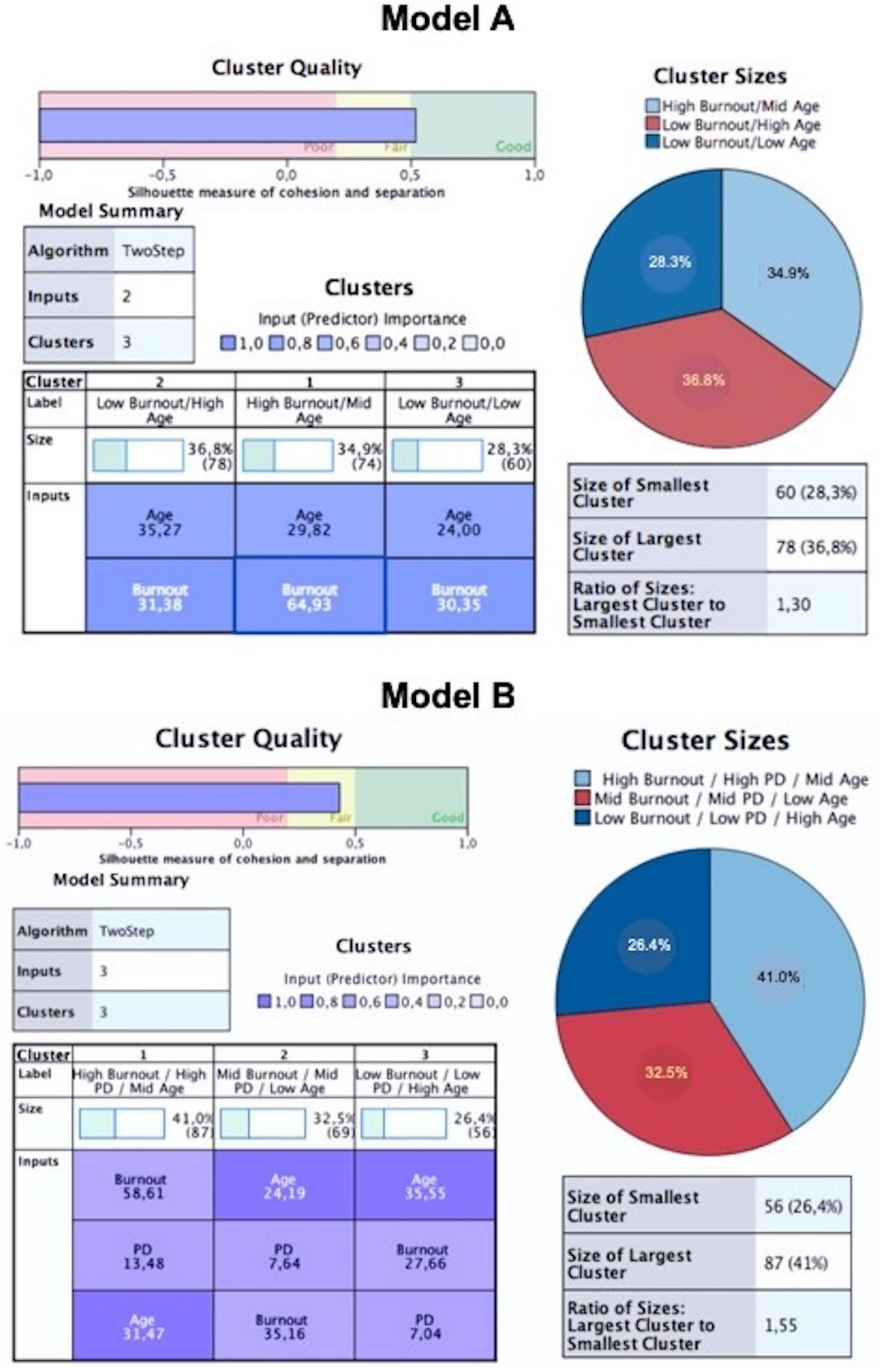
Cluster modelling. Detailed information for both Cluster Models. Variables included: Age (group), MBI-based burnout scores (Model A); Age (group), Psychological disturbance (PD) and MBI-based burnout scores (Model B).

Through a two-step cluster analysis, the optimum number of clusters for the combination of two continuous variables (i.e. Burnout, age) was determined (Fig 1). Starting from fifteen possible clusters, an adequate combination of the factors was found for the solution of three different profiles (i.e. Silhouette measure of cohesion and separation near 0.55-good fit), according to the scores registered in the variables of correctional officers:

*Cluster 1*: Higher Burnout ($\bar{x}$ = 64.93;*SD* = 12.3) and mid age ($\bar{x}$ = 29.82;*SD* = 5.0), containing 34.9% of the valid sample.

*Cluster 2*: Lower Burnout ($\bar{x}$ = 31.38;*SD* = 13.0) and older age ($\bar{x}$ = 35.27;*SD* = 3.9), containing 36.8% of the valid sample.

*Cluster 3*: Lower Burnout ($\bar{x}$ = 30.35;*SD* = 12.1) and younger age ($\bar{x}$ = 24.00;*SD* = 2.7), containing 28.3% of the valid sample.

#### Mean comparisons for Model-A

Through mean comparisons, conducted by means of the One-Way ANOVA for Psychological Disturbance (PD) (*F*_(2,209)_ = 13.641;*p*<0.001), frequency of alcohol consumption (FA) (*F*_(2,184)_ = 4.786;*p* = 0.009) and frequency of physical exercise (FPA) (*F*_(2,187)_ = 9.200;p<0.001), a set of significant differences was found between correctional officers belonging to different clusters, distributed according to age and MBI sum score of Burnout. Furthermore, through the conduction of Post-Hoc analyses (Tukey) for determining significant mean differences between specific clusters, it was found that: Regarding PD, guards belonging to Cluster 1 (high burnout/mid age profile) present significantly higher scores than those belonging to Clusters 2 (low burnout/older age) and 3 (low burnout/younger age). The frequency of alcohol consumption is significantly lower for participants who are in Cluster 2 (low burnout/older age) compared to the other two Clusters, characterized by lower mean values of age but different values of Burnout. Finally, and regarding the reported frequencies of physical exercise, correctional officers belonging to Cluster 3 (low burnout/younger age profile) present significantly higher frequencies than those who are older, despite the scores observed in Burnout, as shown in Table 4.

**Table 4:** Multiple comparisons (Tukey HSD). Factor: Two Step Cluster (Model A).

Dependent Variable	(I) Cluster		(J) Cluster	Mean Difference (I-J)	Std. Error	Sig.	95% CI	
							Lower	Upper
Psychological Disturbance	1	>	2	3.01*	0.69	0.000	1.38	4.64
		>	3	3.39*	0.73	0.000	1.66	5.14
	2	<	1	-3.01*	0.69	0.000	-4.64	-1.38
		>	3	0.38	0.73	0.857	-1.33	2.11
	3	<	1	-3.40*	0.74	0.000	-5.14	-1.66
		<	2	-0.38	0.73	0.857	-2.11	1.33
Frequency of Alcohol Consumption	1	>	2	0.21*	0.08	0.032	0.01	0.41
		<	3	-0.03	0.09	0.936	-0.24	0.18
	2	<	1	-0.21*	0.08	0.032	-0.41	-0.01
		<	3	-0.24*	0.09	0.017	-0.45	-0.04
	3	>	1	0.03	0.09	0.936	-0.18	0.24
		>	2	0.24*	0.09	0.017	0.04	0.45
Frequency of Physical Activity	1	<	2	-0.13	0.13	0.574	-0.44	0.18
		<	3	-0.56*	0.14	0.000	-0.89	-0.24
	2	>	1	0.13	0.13	0.574	-0.18	0.44
		<	3	-0.43*	0.13	0.005	-0.75	-0.11
	3	>	1	0.56*	0.14	0.000	0.24	0.89
		>	2	0.43*	0.13	0.005	0.11	0.75

*. The mean difference is significant at the 0.05 level.

#### Cluster Model-B

Through a two-step cluster analysis, the optimum number of profiles was determined for the combination of three continuous variables (i.e. Burnout, Age and Psychological Disturbance-PD), as shown in Fig 1. Starting from fifteen possible clusters, an adequate combination of factors for the solution of three different clusters was found (i.e. Silhouette measure of cohesion and separation of 0.46-fair fit), according to the scores registered for these variables in the study sample of correctional officers:

*Cluster 1*: Higher PD ($\bar{x}$ = 13.48;*SD* = 4.0), high Burnout ($\bar{x}$ = 58.60;*SD* = 17.3) and mid age ($\bar{x}$ = 31.47;*SD* = 5.3), which corresponded to 41.0% of the valid sample.

*Cluster 2*: Middle PD ($\bar{x}$ = 7.67;*SD* = 2.7), mid Burnout ($\bar{x}$ = 35.15;*SD* = 15.7) and lower age ($\bar{x}$ = 24.19;*SD* = 2.7), corresponding to 32.5% of the valid sample.

*Cluster 3*: Lower PD ($\bar{x}$ = 7.04;*SD* = 2.8), lower Burnout ($\bar{x}$ = 27.66;*SD* = 11.6) and higher age ($\bar{x}$ = 35.55;*SD* = 3.3), containing 26.4% of the valid sample.

#### Mean comparisons for Model-B

When including PD as a combining variable for building the Cluster Model B, and comparing (by Analysis of Variance) the scores of correctional officers obtained in different study variables according to their burnout profile, age and PD, a set of significant differences was found for: frequency of alcohol consumption (*F*_(3,184)_ = 3.560;*p* = 0.030), and subjective social support (*F*_(2,184)_ = 10.484;*p*<0.001). Through Tukey’s Post-Hoc analysis, it was determined, in the case of specific binomial comparisons, that: First, regarding alcohol consumption, correctional officers located in Cluster 1 (high PD/ high burnout/ mid age) have a significantly higher frequency of this than subjects who belong to Cluster 3 (low PD/ low burnout/ older age profile). Secondly, in the case of subjective social support (SS), it was found that guards in Cluster 3 ($\bar{x}$ = 30.96;*SD* = 2.61) have a higher perceived social support than those in Cluster 1 ($\bar{x}$ = 27.73;*SD* = 3.65). Differences were also significant when comparing Cluster 1 (high PD/ high burnout/ mid age) with Cluster 2 (mid PD/ mid burnout/ lower age), in which the first group (characterized for having comparatively higher scores in burnout and PD) showed significantly lesser means of perceived social support, as presented in Table 5.

**Table 5:** Multiple comparisons (Tukey HSD). Factor: Two Step Cluster (Model B).

Dependent Variable	(I) Cluster		(J) Cluster	Mean Difference (I-J)	Std. Error	Sig.	95% CI	
							Lower	Upper
Frequency of Alcohol Consumption	1	>	2	0.05	0.083	0.817	-0.15	0.25
		>	3	.230*	0.088	0.027	0.02	0.44
	2	<	1	-0.05	0.083	0.817	-0.25	0.15
		>	3	0.179	0.091	0.121	-0.04	0.39
	3	<	1	-.230*	0.088	0.027	-0.44	-0.02
		<	2	-0.179	0.091	0.121	-0.39	0.04
Social Support	1	<	2	-2.83*	0.48	0.000	-3.96	-1.69
		<	3	-3.23*	0.51	0.000	-4.43	-2.02
	2	>	1	2.83*	0.48	0.000	1.69	3.96
		>	3	-0.40	0.53	0.736	-1.66	0.86
	3	>	1	3.23*	0.51	0.000	2.02	4.43
		<	2	0.40	0.53	0.736	-0.86	1.66

*. The mean difference is significant at the 0.05 level.

## Discussion and conclusions

The first aim of this study was to characterize the burnout profile of correctional officers. In this regard, we hypothesized that correctional officers have differential burnout profiles, related to demographic factors and mental health indicators. Globally, and in a descriptive sense, this research found that MBI indicators of Burnout [1,66] show relatively lower scores if compared to other occupational target groups considered psychosocially vulnerable by different empirical studies dealing with Colombian workers [68,71]. However, it is true that working conditions of correctional officers substantially vary from those other studied professions (e.g. professional drivers, professors, physicians), and it makes worthy not only studying raw scores on psychometric scales, but the associations and mechanisms between, in this case, burnout, individual features and health of workers. In this sense and, bearing in mind the found associations between study variables, the entire set of correlations of MBI factors were coherent to the reported in the aforementioned research experiences. For instance, EE and CY kept a positive reciprocal association, and a negative correlation with the PE. It is interesting to consider the negative association between EE, CY and the frequency of physical activity reported by correctional officers, and the positive associations between EE and PD scores. In fact, PD (also known as *Psychological Distress*) has been associated, through cross-sectional and, even, longitudinal studies, with the presence of burnout in different groups of workers [72,73].

Another interesting finding that should be discussed is (although not related to EE and CY) the positive association between officers’ rank and PE; even when this finding is based in a bivariate correlation and we cannot infer causality, it may be explained by the differences in the work structure: other studies have stated that workers in higher hierarchical positions, that may allow a greater perception of control at work and decision making, usually present higher scores on variables related to personal accomplishment at job [74,75]. In this regard, and bearing in mind both this rationale and the linear relationship between age/tenure and rank, suggesting a greater decision latitude and discretion among older guards, it was not surprising to find higher scores of PE in this segment of the sample.

As for the prevalence of different health issues reported by Colombian correctional officers, it is noteworthy that, although the percentages obtained for health problems such as high cholesterol (11.3%), hypertension (4%), cardiovascular disease (3.9%), ergonomic illnesses (2.6%) and diabetes mellitus (2%) do not substantially exceed the prevalence reported by other different groups of Colombian workers [75,76], these rates acquire potentially negative implications for the occupational health of this population. This is especially true when considering that the mean age of the sample was only $\bar{x}$ = 30.18 years. From a prospective approach, these data could explain: a) the prevalence of burnout, positively associated with the seniority of employment, as described in other studies with Latin American correctional officers [7], and b) a substantial increase of illness prevalence rates over time. At the same time, this fact implies the importance of designing and implementing health training programs for this particular population, focused on the identification of risks, symptoms, healthcare sources and strategies, as suggested for different high-risk professions [21,29, 77,78].

The second objective of this study was to associate the burnout profile of correctional officers to their health and lifestyle factors, hypothesizing in accordance with some relationships between burnout, age and physical/mental health issues reported by workers in different previous empirical experiences [3,5,14,29] that high-burnout profiles will show less favorable health and lifestyle outcomes. In this regard, although both cluster models (A and B) showed a similar trend for age distribution (i.e. mid and high values for higher burnout rates), the model B associated a greater PD with higher burnout profiles.

In the case of mean comparisons, it was found that correctional officers who experienced a burnout (model A) and burnout + PD (model B) showed: riskier frequencies of alcohol consumption (higher) and physical activity (lower) in the case of model A, and a riskier frequency of alcohol consumption (higher), overweight rates (higher) and social support (lower) for model B. In short, it is important to highlight some relevant findings of this research for what concerns this: first, that both a higher level of alcohol consumption and the fact of being overweight (and other health issues) were related to more psychosocial hazards at work, especially among workers belonging to high-risk professions such as prison-related ones [28,33,79]; and secondly, that other studies have related job stress and burnout to lower rates of social support, and have remarked the importance of strengthening co-workers’ support as a way of coping with adverse factors at the workplace [16,20].

As an overall balance, it was quite interesting to find how correctional officers reporting higher scores on burnout are those that trendily present a more unfavorable set of lifestyle factors: higher alcohol consumption, less physical activity and more PD for the model A, and higher alcohol consumption and less social support for the model B, when introducing PD as a clustering factor. In this regard, studies as the performed by Kuhn and Flanagan [80] have remarked the need of strengthening self-care issues on working forces prone to show burnout symptoms, based on the need of improving their health and psychosocial environment for preventing negative occupational outcomes, including sickness, absenteeism and mental health problems in the future [45,81].

Further, and considering the observed in the categorical analyses, it is worth to discuss the significant differences found in terms prevalence for two health indicators: hypertension and ergonomic illnesses. In both cases, the association between these health problems and the fact of having higher Burnout scores was significant. Regarding hypertension, different studies have suggested a relationship between Burnout and a higher prevalence of it among workforces experiencing high demands at work. For instance, May et al. [82] found in a sample of young adult females that Burnout in the scholar context was significantly related to elevated ambulatory blood pressure (BP), and Landsbergis et al. [83] positively associated hypertension to job strain -closely related to burnout- among workers of eight different worksites of the United States. Secondly, and as for ergonomic illnesses, empirical studies have related different sources of work stress with different ergonomic complains, including musculoskeletal pain [84,85], mostly explained by elevated levels of ACTH (adrenocorticotropic hormone) and cortisol that, since the activation is prolonged once individuals are constantly exposed to work stressors, may turn into chronic pain or, even, cause work disability when not properly diagnosed and treated [86,87].

Also in this sphere (i.e. health complains related to Burnout), several studies have also discussed the relationship between working conditions, physical and mental health indicators and work-related outcomes of many of the so-labeled *vulnerable* occupational groups [9,88,89]. In the same way as some previous studies [47], the present research highlighted the negative impact of burnout on factors such as job performance, job satisfaction and interference with other relevant life spheres and activities like family, leisure and recovery [7,90].

Furthermore, some specific components of health, such as individual habits (e.g. sleep, alcohol consumption, physical exercise), have been characterized as critical for coping with work stressors [27,91]. As observed in this study, there is a high prevalence of correctional officers who present adverse health habits, such as high physical inactivity (48%). These proportions appear to be relatively high in comparison with other occupational groups [14,91,92], factor which should also be addressed in order to promote the occupational health of workers, especially in jobs with high prevalence of sedentary lifestyles [91,93], such as the case of prison officers [94,95].

### Practical implications

Finally, we should mention the importance of strengthening occupational programs aimed at reducing objective hazardous working conditions (and their subjective impact), that contribute to the development of burnout and, at the same time, of different health complains or adverse conditions among workers. Although most of them are related to the penitentiary environment, this problem could be managed from the perspective of occupational health and safety, with the aim of preventing potentially stressful circumstances for prison guards, such as the long working hours [7,88,89], excessive demands [48], and even the possibility of suffering aggressions or violence while working [96]. Concretely linking our results with possible applications for the occupational health of correctional officers, this study highlighted the need of strengthening three essential spheres: firstly, intervention programs on psychosocial factors at work should consider the specific stress sources of correctional officers’ job; it is common to find unsuccessful interventions based on programs designed for other professions, whose characteristics and dynamics may substantially vary from the prison environment. While (from a global perspective) the existence of certain types of job entails higher psychosocial risks, if compared to other professions, most of the effective programs oriented towards the management of stress and burnout constitute a necessary first approximation to a series of actions that, progressively and systematically [97], should be implemented in order to prospectively improve the health and well-being of the most vulnerable occupational groups.

Secondly, we described how burnout may differentially affect officers in accordance to variables such as age/tenure, rank, mental health indicators and perceived social support; these factors must be kept in mind for considering the inclusion of specific components aimed at strengthening these spheres on potential beneficiaries of interventions.

Finally, and specifically regarding lifestyle factors, it is worth remarking its proven value for both physical and mental health. However, both our results and other previous experiences suggest that the strengthen of physical activity could have an additional (plus) usefulness, i.e., contributing in the decreasing of workers’ Burnout, as suggested by other researches [88,98], all framed in the importance of improving their health and welfare.

### Limitations of the study

Although the questionnaires used in this research have a proven good reliability, they are still vulnerable to the self-report bias, typical of cross-sectional studies [99]. The evidence has shown that self-report measures can produce different biases which may vary from social desirability to a substantial lack of sincerity [100], especially considering that participants completed a questionnaire at the workplace and that it included potentially “sensitive" issues such as alcohol consumption and job performance, and in such a situation the veracity of the answers in self-report studies tends to be usually low [101]. Furthermore, although there are different research experiences that provide a theoretical explanation for some health issues of workers based on the presence of burnout, this cross-sectional design did not allow us to infer causality from the association between the burnout and health outcomes of correctional officers.

Moreover, and keeping in mind the root and theoretical assumptions of the Burnout’s behavioral/emotional components, we believe that its diagnosis should be made with more sources of information than the sole MBI, since it is necessary -but not sufficient- for this task. In this sense, it is suggestible to use supplementary information for studying health indicators and adverse working conditions of workers that may be, for instance, undiagnosed or underestimated by them (e.g. reviewing objective occupational and clinical reports, always keeping in mind ethical considerations and procedures).

Thirdly, we should state that this is not a prospective study; for this reason, the reported rates of prevalence of illnesses may vary (increase) with the passing of time and with the experience of new potential stressors at work, thus leading to new burnout/health complain predictors, keeping in mind the relatively low mean of age ($\bar{x}$ = 30.18) of the sample. Additionally, the low female representation in this occupational group (very concordant to the obtained sample) makes it difficult to design studies focused on gender differences or more reliable comparisons based on statistical parameters.

Finally, as for the geographical distribution of the sample, the working conditions of officers may vary depending on variables such as rewards perceived at work, size and type of population of the correctional establishment in which they work, and other specific conditions that are difficult to consider in cross-sectional studies that are based on convenience sampling.

## Supporting information

S1 Dataset Raw data is available in the file (database) attached to the electronic version of this manuscript. (SAV)

## Abbreviations

EE: Emotional Exhaustion
CY: Cynicism or depersonalization
PE: Professional Efficacy
PD: Psychological Disturbance
SS: Social Support
FA: Frequency of Alcohol Consumption
FPA: Frequency of Physical Activity or Exercise
